# Prescription of Rifampicin for *Staphylococcus aureus* Infections Increased the Incidence of *Corynebacterium striatum* with Decreased Susceptibility to Rifampicin in a Hungarian Clinical Center

**DOI:** 10.3390/pathogens12030481

**Published:** 2023-03-18

**Authors:** László Orosz, György Lengyel, Klára Makai, Katalin Burián

**Affiliations:** 1 Department of Medical Microbiology, University of Szeged, H-6725 Szeged, Hungary; 2 Infection Control Department, Semmelweis University, H-1085 Budapest, Hungary; 3 Central Pharmacy of Albert Szent-Györgyi Health Center, University of Szeged, H-6725 Szeged, Hungary

**Keywords:** *Corynebacterium striatum*, antimicrobial resistance, rifampicin, IR Biotyper^®^

## Abstract

Several reports have suggested a role for *Corynebacterium striatum* as an opportunistic pathogen. The authors have conducted a retrospective study at the Clinical Center of the University of Szeged, Hungary, between 2012 and 2021 that revealed significantly increased rifampicin resistance in this species. This work aimed to investigate the reasons behind this phenomenon. The data were collected corresponding to the period between 1 January 2012 and 31 December 2021 at the Department of Medical Microbiology, University of Szeged. To characterize the resistance trends, the antibiotic resistance index was calculated for each antibiotic in use. Fourteen strains with different resistance patterns were further analyzed with Fourier-transform infrared spectroscopy using the IR Biotyper^®^. The decline in *C. striatum* sensitivity to rifampicin seen during the COVID-19 pandemic may have been attributable to the use of Rifadin^®^ to treat concomitant *Staphylococcus aureus* infections. The fact that the IR Biotyper^®^ typing method revealed that the rifampicin-resistant *C. striatum* strains were closely related supports this hypothesis. The IR Biotyper^®^ infrared spectroscopy proved to be a modern and fast method to support effective antimicrobial stewardship programs.

## 1. Introduction

Corynebacteria are pleomorphic, non-spore-forming bacteria that can range in morphology from a club to long, slender bacilli. These microorganisms are common, especially in soil and water, and some of them are naturally present on human skin and mucous membranes as commensal flora. Over a hundred species have been discovered to date, and fifty or more of these are linked to illnesses in humans [1,2,3].

*Corynebacterium striatum* (*C. striatum*) plays a unique and debatable role among these. Since this species was initially identified as a bacterium with the potential for disease in the 1980s, it has come to be recognized as an opportunistic pathogen, particularly but not only in immunosuppressed individuals [3,4,5]. Numerous nosocomial infections have shown *C. striatum* to be a factor. The bulk of these initial cases has been restricted to endocarditis, isolated bacteremia, central line infections, and respiratory tract infections [6,7,8,9]. The focus has been expanded to encompass a wide range of additional illness types [9,10,11].

*Corynebacterium striatum* has been implicated in several nosocomial infections. The majority of these affect the respiratory tract, the bloodstream, and the endocardium, on rare occasions [6,7,8,12,13,14]. Acquired resistance to β-lactam antimicrobials, clindamycin, erythromycin, ciprofloxacin, and gentamicin is characteristic of multidrug-resistant *Corynebacterium* species spreading in the hospital environment [15]. Confirmed rifampicin resistance in this species is variable, mainly driven by local specificities [16,17]. Unfortunately, the literature is scarce on this topic, and it is limited to specific countries.

On the other hand, increasing resistance is predicted to be a major issue for the genus in the future [17]. The multidrug resistance of the *Corynebacterium* genus is a further cause for concern. Clindamycin, erythromycin, ciprofloxacin, and gentamicin resistance are impacted by the spread of multidrug-resistant *Corynebacterium* species in the hospital environment [15]. The current effective medications against *C. striatum* clinical isolates are glycopeptides, linezolid, quinupristin/dalfopristin, daptomycin, and tigecycline [18,19,20]. Unfortunately, there is little literature that is not exclusive to one or two nations. In the future, the genus is predicted to face significant challenges because of the rising resistance [18].

Additionally, *C. striatum* strains can produce biofilms, which dramatically increases their pathogenicity. Several bacterial mechanisms that affect virulence and resistance are made easier by the structure known as a biofilm. These include the ability to adhere, exchange of metabolites, cellular communication, resistance to antimicrobials, and thwarting of host immunity. Thus, the development of biofilms enhances infection and colonization. In the presence of invasive medical devices, such as catheters and endotracheal tubes, the *C. striatum* strains become more aggressive. As a result, the development of bacterial biofilms raises medical expenses, prolongs hospital stays, and possibly spreads antibiotic-resistance genes [21,22,23,24].

Despite this, *C. striatum* infections of the respiratory system have long been a source of debate. However, a growing number of articles has recently revealed its pathogenicity in the respiratory tract, particularly in immunocompromised patients [3,4,25]. During the years of the COVID-19 pandemic, the number of these patients has risen considerably [26,27,28,29]. In the context of COVID-19, several reports have suggested a role for *C. striatum* [4,17,30,31]. The authors have also conducted a retrospective study at the Clinical Center of the University of Szeged, Hungary, between 2012 and 2021 to determine the prevalence and resistance pattern of this species [17]. As a result, we have stated that rifampicin resistance significantly increased in the *C. striatum* isolates during this period. Due to the suppression of bacterial RNA polymerase, rifampin is bactericidal [32]. Its primary indications are the treatment of infections, particularly tuberculosis and leprosy, and meningococcal prevention [32]. However, there is evidence that the use of rifampicin–minocycline- or rifampicin–miconazole-impregnated catheters reduces the incidence and costs of catheter-related bloodstream infections [33]. In addition, there are limited studies investigating the combination of rifampicin and colistin for the treatment of multidrug-resistant *Acinetobacter baumannii*. As the COVID-19 pandemic caused serious concerns about nosocomial outbreaks of *A. baumannii* worldwide [28,34,35,36,37], including the clinical center in Szeged, Hungary (unpublished data), we hypothesized that the amount of rifampicin administered to prevent catheter-associated infections may have increased during this fight.

Recently, the IR Biotyper^®^ (Bruker GmbH, Bremen, Germany), an automated typing system based on Fourier-transform infrared spectroscopy (FTIR) became commercially available. FTIR spectroscopy is a spectrum-based approach that assesses the infrared light absorption by chemicals present in the bacterial cell. The resulting infrared spectrum gives a unique fingerprint that is reflective of the proteins, lipids, and carbohydrates of the bacterial cell. Consequently, each bacterium has a very distinct infrared absorption pattern that allows subspecies-level identification [38,39]. Despite its ease and quickness, IR Biotyper^®^ has not yet been used to analyze *C. striatum* strains.

This work aimed to investigate the reasons behind this phenomenon using standard statistical and state-of-the-art typing methods to provide data for effective antimicrobial stewardship programs.

## 2. Materials and Methods

### 2.1. Study Setting

The data were collected corresponding to the period between 1 January 2012 and 31 December 2021 at the Department of Medical Microbiology, University of Szeged. Data collection was performed electronically in the records of the laboratory information system corresponding to samples positive for *C. striatum*. The data were exported from the clinical microbiology laboratory information system (MedBakter, Asseco Central Europe Ltd., Budapest, Hungary) and were reported into a customized database. The data included the sex, age, submitting diagnosis of the patients, types of specimens, name of the sending department, and antimicrobial susceptibility patterns. Antimicrobial susceptibility testing results were determined and interpreted according to the European Committee on Antimicrobial Susceptibility Testing (EUCAST) [40].

### 2.2. Calculating Antibiotic Resistance Index

To calculate the ARI, the model for measuring antibiotic resistance used by De Socio et al. was followed [41]. Briefly, for each antibiotic tested, a score of 0 for susceptibility, 0.5 for intermediate resistance, or 1 for resistance was assigned, and the ARI was calculated by dividing the sum of these scores by the number of antibiotics tested, giving a maximum score of 1.

### 2.3. Strain Typing with IR Biotyper^®^ (IRBT)

Unfortunately, only 14 strains were available for this type of analysis. Two of these were from 2021 and the rest from 2022. All typed *C. striatum* isolates were cultured at 37 °C for 24 h on Mueller–Hinton Agar (MHA; Bio-Rad, Hercules, California, USA). The first step was to collect a loopful of bacterial culture (~1 µL) and suspend it in 50 µL of 70% ethanol in a 1.5 mL microcentrifuge tube with sterile metal rods provided by the kit manufacturer. Using a vortexer, a uniform suspension was achieved. A 50 µL volume of sterile water was added after 1 min of vortexing, and the remaining 100 µL of the solution was vortexed again for 1 min. Then, 12 µL of the two infrared (IR) test standard 1 (IRTS1) and IR test standard 2 (IRTS2) suspensions were spotted onto the IRBT silicon plate with 15 µL of the bacterial suspension and dried at 37 °C for 30 min until a film was formed from the drops. The dried silicon plate was then put into the IRBT spectrometer (Bruker Daltonics GmbH & Co. KG, Bremen, Germany) with the analytical parameters at their defaults. OPUS 7.5 software was used to collect the isolates’ spectra (Bruker Daltonics GmbH & Co. KG, Bremen, Germany). The spectra that met the default quality criteria of absorption [0.4 arbitrary unit (AU) < D value < 2 AU], signal/noise (<150 × 10^−6^ AU), signal/water (<300 × 10^−6^ AU), and fringes (<100 × 10^−6^ AU) were determined as “quality pass” in the IRBT analysis. To create the 2D scatter plots and dendrograms, the spectra acquired with “quality pass” were used. The software includes a function that automatically suggests a cut-off value that establishes the minimum distance at which two spectra are regarded as belonging to the same cluster.

### 2.4. Data Analysis

The data were exported from the laboratory information system into the MS Excel 2016 (Microsoft Corp., Redmond, WA, USA) and GraphPad Prism version 8 (GraphPad Software, San Diego, CA, USA) software. MS Excel 2016 was used to store the data and to determine the different antibiotic resistance index (ARI) curves. GraphPad Prism 8 was used for statistical analysis and plotting. All values are expressed as means and ranges, where appropriate. The *p* values < 0.05 were considered statistically significant. The Pearson correlation function of GraphPad Prism 8 was used to determine the correlation between the amounts of rifampicin used and the ratio of resistance.

Annual consumption data for rifampicin-containing products were exported from the database of the Central Pharmacy of the Clinical Center. The Q-Q plot function of GraphPad Prism 8 was used to test the normal distribution of all used variables.

## 3. Results

### 3.1. The C. striatum Was Isolated Mainly from the Respiratory Tract of Elderly Male Patients with COVID-19

**Figure 1:**
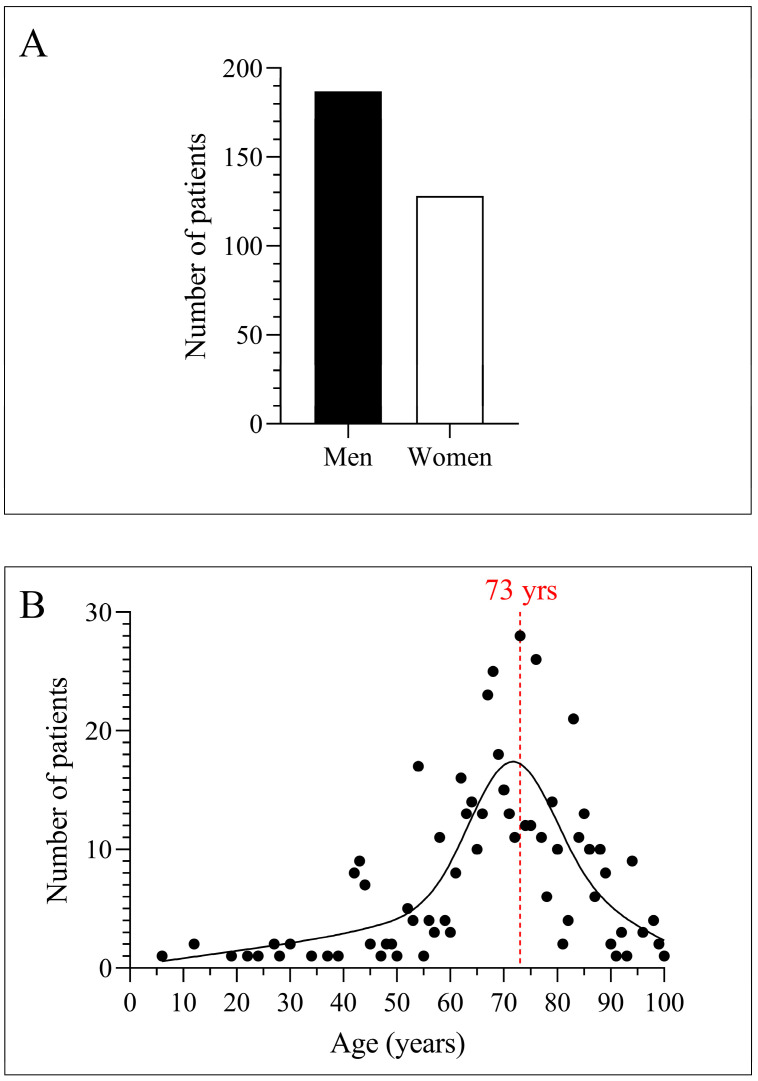
Distribution of study participants by gender and age. (**A**) Patient distribution by gender. (**B**) Age distribution of patients.

**Figure 2:**
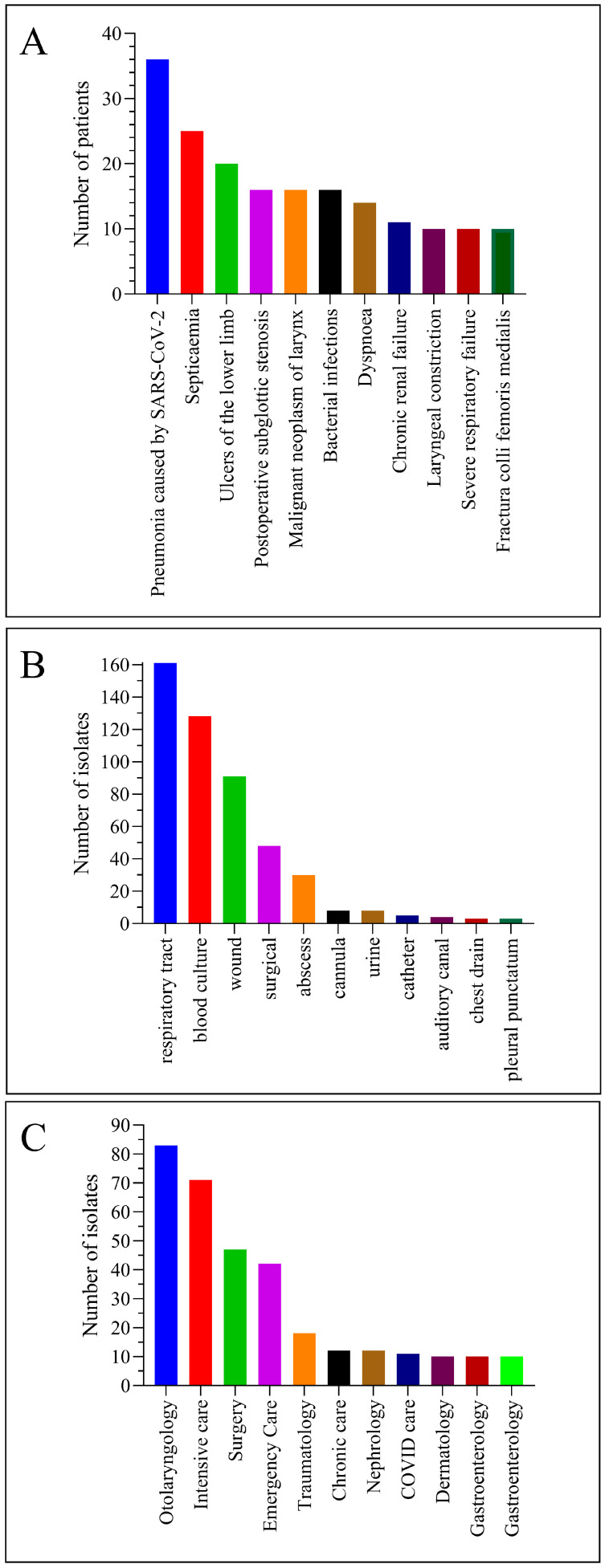
Important clinical and epidemiological patient characteristics. (**A**) Most frequent referring diagnoses. (**B**) Most prevalent specimen types. (**C**) Most common departments sending positive specimens.

During the study period, we isolated *C. striatum* from a total of 315 patients. Of these patients, 187 were male (59%) and 128 were female (41%) (Figure 1A). The age distribution was dominated by older people. The most common age (mode of the data series) was 73 years (Figure 1B). The most common patient diagnosis was pneumonia caused by SARS-CoV-2. This was followed by septicemia, ulcers of the lower limb, and postoperative subglottic stenosis (Figure 2A). Most strains were isolated from the respiratory tract and blood cultures. This was followed by isolates from the wound, surgical, and abscess origin (Figure 2B). The department that submitted the greatest number of positive samples was otolaryngology. Afterward, intensive care, surgery, and emergency care followed (Figure 2C). 

Therefore, *C. striatum* was mainly isolated from the respiratory tracts of elderly male COVID-19 patients.

### 3.2. The Number of C. striatum Isolates, Their Resistance to Rifampicin, and the Use of Certain Antibiotics Containing Rifampicin Increased Concomitantly during the Study

**Figure 3:**
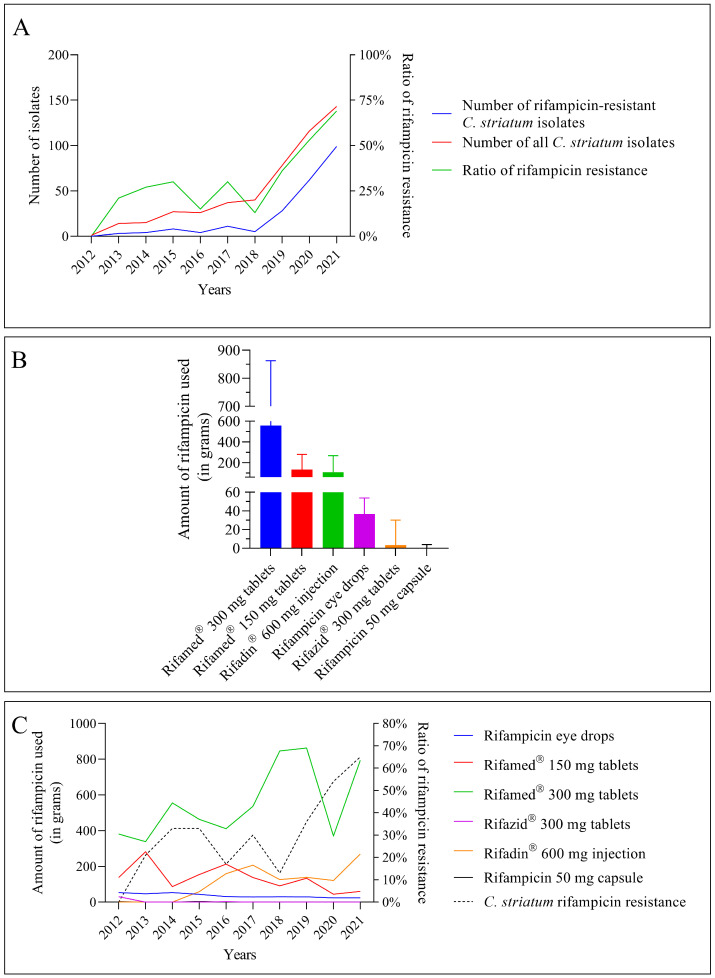
Trends in the usage of rifampicin-containing formulations and rifampicin resistance in *C. striatum* from 2012 to 2021. (**A**). Trends in rifampicin resistance among *C. striatum* isolates. (**B**). Quantities of various rifampicin-containing medications used (columns indicate annual mean with range). (**C**). Dynamics of rifampicin resistance and the usage of various formulations.

Since 2018, the number of *C. striatum* isolates in the clinical center has increased dramatically (Figure 3A, red line). The rise of rifampicin resistance is also noteworthy (Figure 3A, blue line). This can be seen in the remarkable growth in the fraction of resistant strains, as well as in absolute terms (Figure 3A, green line). From all these data, rifampicin resistance was remarkably increasing in *C. striatum* isolates over the period studied.

The usage of rifampicin-containing medications in the clinical center during the period under consideration was the subject of the following section of our research. Rifamed^®^ pills were utilized at the greatest rate, as can be observed (Figure 3B). Rifadin^®^ injection was the next-most-often used formulation. Rifazid^®^ pills and rifampicin capsules were supplied in small numbers.

The second stage of our research looked at the relationship between medication usage and the spread of rifampicin resistance over time. The usage of various medications revealed a highly diverse scene (Figure 3C). This graph also shows that Rifamed^®^ 300 mg pills were the most utilized in the clinical center, with usage rising from 2017 to 2020 (Figure 3C, green line). Following the fall in 2020, there was a sharp surge in 2021. The usage of Rifadin^®^ 600 mg injectable has also increased since 2016 (Figure 3C, orange line). In 2017, and especially in 2021, further big rises are projected. Throughout the study, the utilization of Rifamed^®^ 150 mg pills fell steadily (Figure 3C, red line). The other medications were used in smaller quantities. One of them was a topical agent (eye drops, Figure 3C, blue line). The rate of rifampicin resistance has been seen to vary, which corresponds to variation in the usage of each medicine (Figure 3C, black dashed line). However, from 2018 forward, there has been a tremendous surge in rifampicin resistance, which is expected to reach 60% by 2021 (more than 60 percent of isolates were resistant to rifampicin).

During the study, the number of *C. striatum* isolates, their resistance to rifampicin, and the use of antibiotics containing rifampicin all increased simultaneously.

### 3.3. Not All Rifampicin-Containing Medications Experienced a Rise in Usage as the Resistance Increased

**Figure 4:**
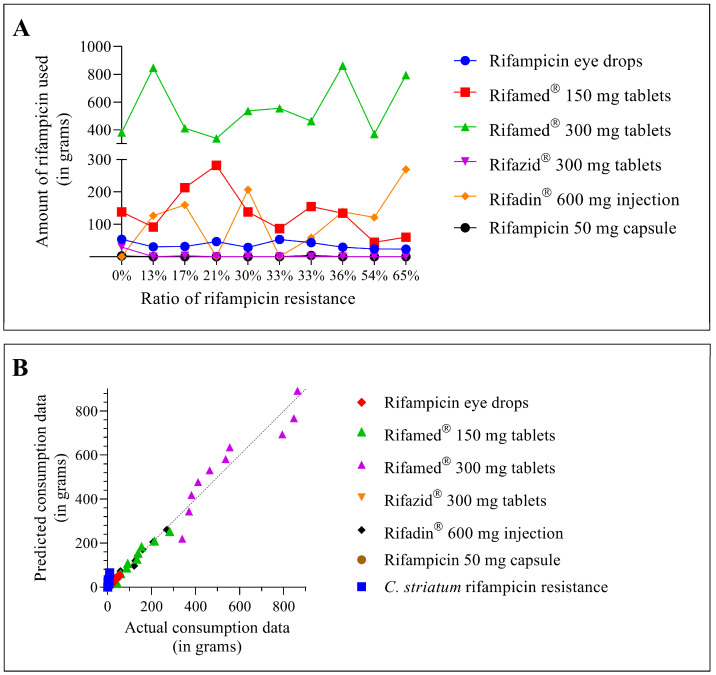
Relationship between rifampicin-containing drug use and rifampicin resistance (**A**) and statistical analysis of the distribution of these variables (**B**). (The 33% resistance ratio occurred in two different years, which is why it is shown twice in (**A**) with the drug use data for that year.)

In the next step, we examined how the use of each agent related to the increase in rifampicin resistance (Figure 4A). The graph clearly shows that the use of each agent has been consistent across different rifampicin-resistance conditions. There was also no significant increase in the doses used. Drugs that were used in higher amounts with low resistance levels (e.g., Rifamed^®^ 300 mg tablets) were used at almost the same intensities for the duration of the study. When resistance increased, the rarely used preparations remained rather stable at their lower doses (e.g., rifampicin eye drops or rifampicin 50 mg capsule). Only two of the six drugs examined showed a change in the trend of use. The use of Rifamed^®^ 150 mg tablets has steadily declined, while the use of Rifadin^®^ 600 mg injectable formulation gained ground between 2012 and 2021 (Figure 4A, red and orange lines, respectively).

Following that, we looked at how much each of the agents utilized may have contributed to the rise in resistance. However, before putting this to the test, it was required to see if the variables under investigation had a normal distribution. We utilized the normality test feature of GraphPad Prism 8 to do this. The program depicts the actual Y values on the horizontal axis and the expected Y values (assuming Gaussian sampling) on the Y-axis in this procedure. The points follow a straight line that matches the line of identity if the data were sampled from a Gaussian (normal) distribution. All the variables fit nicely on the normal distribution line, as shown in Figure 4B. As a result, we could apply statistical tests on Gaussian distribution variables.

Therefore, not all rifampicin-containing medications rose in usage as resistance developed, but all variables had a normal distribution.

### 3.4. The Use of Some Rifampicin-Containing Drugs Correlates Better with Increased Resistance Than Others

As a result, the Pearson correlation coefficient was used to evaluate the relationship between the usage of various rifampicin-containing medications and rifampicin resistance (Table 1). Rifadin^®^ 600 mg injectable (r = 0.56) and Rifamed^®^ 300 mg tablets (r = 0.27) had the greatest Pearson’s *r*-values. Thus, certain rifampicin-containing medications have a stronger correlation with resistance than others.

**Figure 5:**
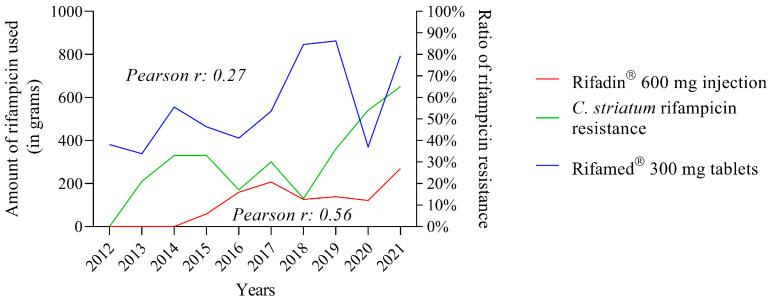
Correlation between the use of Rifadin^®^ 600 mg injection and Rifamed^®^ 300 mg tablets and rifampicin resistance.

When the consumption data from the two formulations are plotted against the evolution of rifampicin resistance in *C. striatum*, the data from the Rifadin^®^ 600 mg injectable formulation, which have a higher Pearson’s *r*-value, follow the evolution of resistance better than the data from the tablet formulation of Rifamed^®^ 300 mg (Figure 5).

### 3.5. The IRBT Analysis Confirms That Rifampicin-Resistant C. striatum Strains Belong to Different Clones Than Sensitive Ones

**Figure 6:**
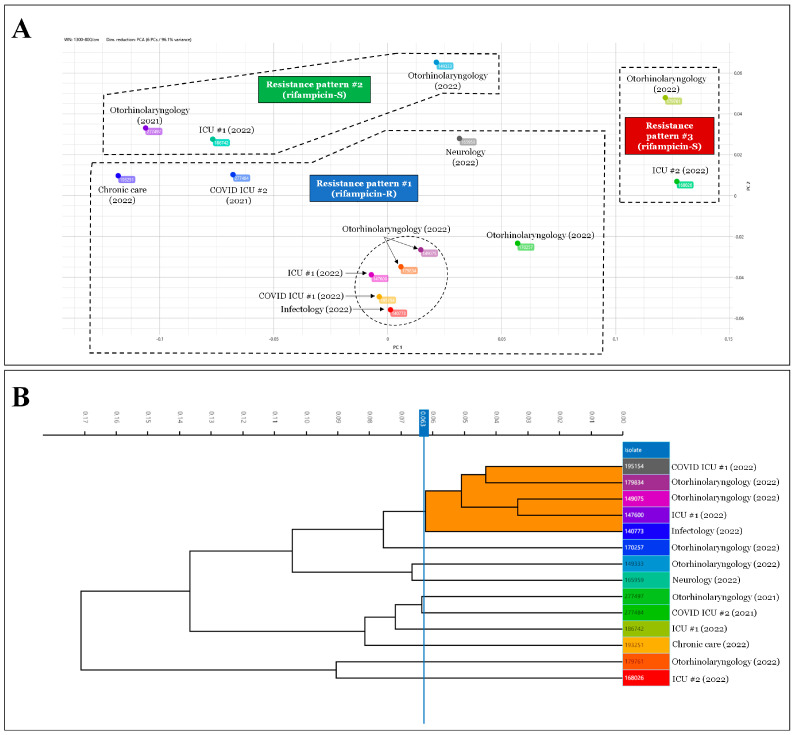
The results of IRBT typing of several *C. striatum* strains. (**A**). The result of the IRBT typing is displayed in 2D scatter plot format. The strains delimited by a dashed line showed the same resistance pattern. Isolates marked with a dashed outline showed the closest relationship on the dendrogram (**B**). (**B**). The result of typing with IRBT is displayed in dendrogram format. Strains highlighted in orange indicate the closest relationship (indicated by a dashed outline in (**A**)).

Comparing all these data with the results of the strain typing, three large clusters could be defined within the 14 strains (Figure 6A). Strains exhibiting rifampicin-resistance patterns were included in the first cluster. The cluster with the most elements (n = 9) is this one. Only one of these strains, from a COVID-19 ICU, was from the year 2021. The second group contained rifampicin-sensitive strains (n = 3) with different resistance patterns. One of these was isolated in 2021. The third group, which had the fewest members (n = 2), included rifampicin-susceptible strains with a different pattern of resistance from the second. Each of these was isolated in 2022. The rifampicin-resistant strains formed a separate cluster that was unrelated to the isolation year, as can be observed from all these data. This was confirmed with a software-generated dendrogram showing the closest relationship between the rifampicin-resistant isolates (Figure 6B, orange cluster; Figure 6A, strains circled with dashed lines). Therefore, the IRBT study revealed that rifampicin-resistant *C. striatum* strains belong to different clones than those that are susceptible.

## 4. Discussion

The nosocomial infections caused by *C. striatum* mostly affect the respiratory tract and the bloodstream [3,12,16,25]. Multidrug resistance in these cases can be a major challenge for clinicians [5,8,11,12,17,42]. Rifampicin is a viable treatment option for catheter-associated infections [43,44]. Therefore, monitoring the resistance of isolates to rifampicin would be an important task for laboratories and antibiotic stewardship programs [17]. 

With these considerations in mind, in the present study, we first investigated the trend in rifampicin resistance among *C. striatum* strains isolated at our clinical center between 2012 and 2021. The patients involved were mostly older men with COVID-19 (Figure 1 and Figure 2). This result is consistent with the literature data. Marino et al. described the case of a 91-year-old patient who was hospitalized with SARS-CoV-2 infection, acquired *C. striatum* bacteremia, and died despite antimicrobial treatment and therapeutic attempts [30]. Yet, statistics from several publications indicate that these are not isolated cases, but instead take the form of hidden nosocomial outbreaks and are often overlooked. Charalampous et al. documented an epidemic of MDR-C. striatum involving 14 COVID-19 patients in three ICUs, which could only be discovered using clinical metagenomics [4]. This makes the diagnosis and distinction of *C. striatum* colonization and infections challenging, particularly in immunocompromised patient groups, such as elderly people with severe COVID-19.

The problem is further complicated by the frequent multidrug resistance of *C. striatum* strains, including agents such as rifampicin, which is commonly used in the treatment of catheter-related bloodstream infections. We also observed an increase in rifampicin resistance in our clinical center, especially after the outbreak of the COVID-19 pandemic [17]. After 2018, a sharp increasing trend emerged, as illustrated in Figure 3A. This can be seen not only in the increase in the absolute number of resistant isolates, but also in the relative proportion, and shows no sign of slowing down in 2021. This worrying phenomenon has recently been described in several publications worldwide [5,12,42,45,46]. The genetic background of this resistance type is primarily acquired resistance because of mutation in the *rpoB* gene, which encodes the β-subunit of bacterial RNA polymerase [47]. As this type of resistance is usually the result of spontaneous mutation, it does not typically spread horizontally [47]. However, it is significantly facilitated by selection pressure caused by the growing usage of rifampicin-containing agents. 

Considering these data, we found it necessary to examine which rifampicin-containing drugs were used in the clinical center during these years and in what quantities. Data from the database of the central pharmacy showed that six such formulations were used during the period under review. These were, in descending order, Rifamed^®^ 300 mg tablets, Rifamed^®^ 150 mg tablets, Rifadin^®^ 600 mg injection, rifampicin eye drops, Rifazid 300 mg tablets, and rifampicin 50 mg capsules (Figure 3B). These formulations cover well the original and magistral formulations of rifampicin available in Hungary [48].

Then, we looked at the annual consumption data per unit mass of each formulation in the following part of our research. We found that the data for Rifamed^®^ 300 mg tablets, which were the most widely used overall, showed remarkable fluctuations (Figure 3C). The increasing trend that had been in place until 2019 was halted in 2020, and the data for 2021 did not reach the high point of 2019. Over the study period, the second-most-used Rifamed^®^ 150 mg pills exhibited a progressively decreasing trend. Data for Rifadin^®^ 600 mg injection also showed a remarkable increase, especially in 2021. Other formulations were used in smaller and more evenly distributed amounts (Figure 3C). To the best of the authors’ knowledge, such a detailed survey has not yet been published in Hungary.

The next step in the study was to investigate how the use of each drug related to different levels of rifampicin resistance. Consumption data of several rifampicin-containing medications are displayed as a function of rifampicin resistance in a dot plot in Figure 4A. The rise of rifampicin resistance did not affect the usage of individual drugs. Agents that were used in large quantities during low-resistance periods were used in almost the same quantities during high-resistance periods. The same is true for agents used at low doses. Only two of the six medications studied demonstrated a shift in use patterns. Between 2012 and 2021, the usage of Rifamed^®^ 150 mg tablets continuously decreased, while the use of Rifadin^®^ 600 mg injectable formulation increased (Figure 4A). However, the authors are convinced that this change was not driven by considerations of spreading resistance. This raises major doubts about whether practitioners’ prescription behaviors reflect local resistance patterns at all. The problem is not unique. In a recent publication, Machowska et al. argued that the absence of suitable diagnostic tools to quickly identify the organism and its antibiotic susceptibility profile, guide antibiotic administration at the point of care, and limit the requirement for wide-spectrum antibiotics is a major factor in erroneous antibiotic prescribing and use [49]. For their part, the authors would add that clinicians do not always seem to follow local resistance trends. Well-functioning local antimicrobial stewardship programs could help to address this problem.

Statistical tests were used to determine which formulations would be linked to an increase in resistance. The Q-Q plot results revealed that all the variables under investigation are linearly positioned on the dashed line, suggesting a normal distribution, as illustrated in Figure 4B. This result determines that the correlations between the different variables can be tested using Pearson’s rank correlation.

In the following step of our research, we looked at how the drug consumption data correlated with the rise in rifampicin resistance. We employed the above-mentioned Pearson’s rank correlation analysis since it is appropriate for data with a normal distribution. Table 1 summarizes the findings of the analysis. The analysis gave positive correlation results for only two agents: Rifamed^®^ 300 mg tablets (r = 0.27) and Rifadin^®^ 600 mg injection (r = 0.56). Correlation coefficients with magnitudes between 0.3 and 0.5 suggest variables with minimal correlation, while those with magnitudes between 0.5 and 0.7 indicate variables that are moderately associated [50]. When the consumption data only for these two agents were reviewed, it was obvious that the time course of the utilization of Rifadin^®^ was most consistent with a rise in rifampicin resistance (Figure 5). The main indications for this medicine are the treatment of tuberculosis and the meningococcal carrier state [51]. However, the application documentation raises the possibility of treating *Staphylococcus aureus* infections with Rifadin^®^. As demonstrated in our previous publication, *S. aureus* was the most frequently co-isolated co-pathogenic bacterium with *C. striatum* in the clinical center between 2012 and 2021 [17]. Based on these findings, it appears reasonable to infer that the selection pressure exerted during the treatment of concurrent *S. aureus* infections may have resulted in *C. striatum* strains losing their rifampicin susceptibility. This assumption is well supported by the fact that the modern Fourier-transform typing procedure showed that these strains were closely related (Figure 6). To the authors’ knowledge, no such typing study has been performed on this species to date, and this further supports the utility of the method in supporting effective antimicrobial stewardship programs. This IRBT method can be useful in characterizing strains with different resistance patterns in the healthcare setting, and thus can be used to facilitate infection prevention and control [52].

## 5. Conclusions

In conclusion, we propose that the reduction in *C. striatum* susceptibility to rifampicin seen during the COVID-19 pandemic at the Clinical Center of the University of Szeged is attributable to the concurrent treatment of *S. aureus* infections with the Rifadin^®^ injectable formulation. The co-occurrence of the two species raises intriguing issues regarding the pathophysiology of respiratory bacterial colonization during COVID-19 infections, which could be a prosperous future study area.

## Figures and Tables

**Table 1:** The detailed results of Pearson’s correlation analysis between rifampicin-containing formulations used between 2012 and 2021 and rifampicin resistance detected in *C. striatum* during the same period and its additional statistics for each comparison. The two highest values are highlighted in bold.

Statistical Indicators	*C. striatum* Rifamicin Resistance Ratio vs. Rifampicin Eye Drops	*C. striatum* Rifamicin Resistance Ratio vs. Rifamed^®^ 150 mg Tablets	*C. striatum* Rifamicin Resistance Ratio vs. Rifamed^®^ 300 mg Tablets	*C. striatum* Rifamicin Resistance Ratio vs. Rifazid^®^ 300 mg Tablets	*C. striatum* Rifamicin Resistance Ratio vs. Rifadin^®^ 600 mg Injection	*C. striatum* Rifamicin Resistance Ratio vs. Rifampicin 50 mg Capsule
Pearson’s *r*-value	−0.5793	−0.5331	**0.2714**	−0.5825	**0.5576**	−0.2639
95% confidence interval	−0.8858 to 0.07929	−0.8705 to 0.1453	−0.4321 to 0.7695	−0.8869 to 0.07441	−0.1109 to 0.8787	−0.7662 to 0.4386
R squared	0.3355	0.2842	0.07364	0.3393	0.3110	0.06964

## Data Availability

Not applicable.
